# Sulpiride-Induced Hyperprolactinemia in Mature Female Rats: Evidence for Alterations in The Reproductive System, Pituitary and Ovarian Hormones

**Published:** 2014-07-08

**Authors:** Sara Mostafapour, Samad Zare, Rajab Ali Sadrkhanlou, Abbas Ahmadi, Mazdak Razi

**Affiliations:** 1 Department of Biology, Faculty of Basic Sciences, Urmia University, Urmia, Iran; 2 Department of Comparative Histology, Urmia University, Urmia, Iran; 3 Department of Anatomy, Faculty of Veterinary Medicine, Urmia University, Urmia, Iran

**Keywords:** Sulpiride, Hyperprolactinemia, Follicular Growth, Atresia, Ovary

## Abstract

**Background:**

The prevalence of hyperprolactinemia following administration of conven- tional antipsychotic drugs requires further investigation. The current study is designed to evaluate the effect of sulpiride (SPD)-induced hyperprolactinemia on alterations to ovarian follicular growth, gonadotropins, and ovarian hormones and to analyze the extent of potential problems in mammary glands.

**Materials and Methods:**

A total of 40 albino Wistar rats were divided into four groups: control (no treatment), control-sham (0.3 ml olive oil), low dose SPD (20 mg/kg) and high dose SPD (40 mg/kg). All compounds were intraperitoneally (IP) administered for a period of 28 days.

**Results:**

After 28 days, we dissected the rats’ ovarian tissues, uterine horns and mammary glands which were sent for histological analyses. We counted the numbers of normal, atretic follicles and corpora lutea (CL). Serum levels of prolactin (PRL), estradiol, progesterone, follicle stimulating hormone (FSH) and luteinizing hormone (LH) were evaluated. SPD-administered animals showed sporadic follicular atresia in different sizes associated with higher numbers of CL on the ovaries. The mammary glands exhibited features of galactorrhea. There was remarkable (p<0.05) elevation in SPD-administered animals’ uterine horn endometrium, myometrium and perimetrium thicknesses. The serum levels of PRL and progesterone significantly (p<0.05) increased, while the serum concentration of estradiol, LH and FSH notably (p<0.05) decreased according to the SPD administered dose. No histological and biological changes occurred in control-sham animals. SPD-induced animals had unsuccessful attempts at mating and decreased pregnancy rates.

**Conclusion:**

The present findings suggest that SPD-induced disturbances depend on PRL level. In addition, an increased PRL level is largely dependent on the administered doses of SPD.

## Introduction

Sulpiride (SPD) is an alternative benzamide compound which selectively blocks postsynaptic dopaminergic neurons. This compound affects the D2 receptors. The drug is widely used as an antipsychotic agent to treat schizophrenia. Similar to other medications of the same classification, SPD causes prolactin (PRL) release (1).

PRL is a polypeptide hormone secreted by the lactotroph cells of the anterior pituitary gland. Release of this hormone is pulsatory with approximately 14 pulses per day and it displays significant daily rhythmic levels (2). The primary physiologic role of PRL is to induce lactation and it interacts with other central nervous system (CNS) and peripheral processes. Its secretion is influenced by both stimulatory and inhibitory endogenous and exogenous substances (2, 3). Antipsychotic drugs are known to result in severe elevations in PRL levels (4-6). A remarkable majority of the patients with psychological problems are treated with conventional antipsychotic drugs such as risperidone. These medications have been shown to significantly elevate serum PRL levels (7, 8). On the other hand, women of reproductive age are susceptible to medical disorders associated with hyperprolactinemia. It is well established that pathologically increased PRL secretion inhibits follicular estradiol production (9), luteal phase defects, polycystic ovary syndrome (10), and severe follicular atresia(10, 11).

In addition to the mentioned disorders, the majority of hyperprolactinemia-induced clinical problems are attributed to the interference of PRL on the hypothalamic-pituitary-gonadal system. PRL suppresses gonadotropin-releasing hormone (GnRH) secretion from the hypothalamus and directly interferes with the pituitary physiologic actions of the gonadotropin luteinizing hormone (LH) and follicle stimulating hormone (FSH) on the gonads (12). On the other hand, follicular growth and granulosa cell physiologic function mainly depend on serum levels of FSH and LH. Therefore dysregulation of ovarian hormones in addition to their impaired correlation with the pituitary gland (feedback mechanisms) will lead to important problems in fertilizing potential (13, 14).

Although conventional antipsychotic drugs are known to elevate PRL levels above the upper limit of normal for both men and women, a reliable estimate for the dose-dependent effect of antipsychotic drug-induced follicular atresia is not readily available. Thus, the present study attempts to reconcile the dose-dependent effect of SPD on follicular growth, hormonal changes and histological structure of uterine horns in rats. Moreover, we have analyzed the mammary glands in order to illustrate hyperprolactinemia-induced histological changes.

## Materials and Methods

### Animals and medication administration methods

This experimental study was approved byUrmia University. In this research, we used 40 mature female Wistar rats, 70 days of age that weighed 160 ± 20 g. The rats were purchased from the Animal Resources Center of the Faculty of Basic Sciences , Urmia University, Urmia, Iran and were acclimatized in an environmentally controlled room (temperature: 20-23˚C and 12 hours light/12 hours dark schedule). Food and water were given ad libitum. In this study all experiments conducted on animals were in accordance with the Urmia University Guidelines of the Ethical Committee for research on laboratory animals. Following a one week acclimation period, the animals were assigned to four groups (n=10 per group): control, control-sham and two test groups. The control group rats received food and water with no treatment. The control-sham group received daily intraperitoneally (IP) injections of 0.3 ml olive oil for 28 continual days. The test subgroups received either daily low dose IP injections of SPD (20 mg/kg) or high dose IP injections of SPD (40 mg/kg), for 28 continual days.

### Histology and morphometry

After 28 days the animals were euthanized by a special CO2 device and the ovarian, uterine horn and mammary gland specimens were dissected out and fixed in 10% formalin fixative for histological investigations, then embedded in paraffin. Serially prepared sections (5-6 μm) were stained with hematoxylin-eosin (Merck Co, Germany). In the ovarian sections, follicles were characterized in terms of their sizes: <100, 101-200, 201-300, 301-400 and >400 μm. Follicular morphology was examined by a microscope with a ×40 objective lens (Olympus, Germany) magnification. Follicles that had a complete layer of flattened granulosa cells, oocytes with cytoplasm, and a normal nucleus were considered normal. Abnormal follicles were classified according to the presence of cytoplasmic damage, a pyknotic nucleus, and combination of damaged nucleus and cytoplasm. Follicular number was estimated by counting the number of follicles in all slides (15). We also counted the corpus luteum (CL) number for each ovary. For histomorphometric analyses, the uterine horn endometrial epithelium, endometrium, myometrium and perimetrium thicknesses were evaluated by a morphometric lens (Olympus, Germany) with a ×40 objective lens. Ultimately the glands’ distribution and numbers per one mm^2^ were estimated.In addition, we assessed the diameters of the mammary gland lobules and histological features.

### Serum sampling and hormonal analyses

Blood samples from corresponding animals were collected directly from the heart. Serum was centrifuged at 3000 g for 5 minutes and subjected to assessments for LH, FSH, progesterone, estrogen (E2) and prolactin levels (PL).

### Radioimmunoassay of PRL, LH and FSH in serum

We added a 100 μl aliquots of sera to the tubes which contained 100 μl labeled hormones with rabbit anti-sera in 0.01 M phosphate buffer (pH 7.6). Anti-rat PRL(Cisbio Bioassays, France) was diluted 1:5000, LH was diluted 1:10000 and FSH was diluted 1:2500. The mixture was incubated for 48 hours at 4˚C. Goat anti-rabbit IgG at a 1:10 dilution (200 μl) was added to the mixture. After remaining for 18 hours at 4˚C, the mixture was subsequently centrifuged at 2000×g for 30 minutes and we used a gamma counter to measure the presence of radioactivity in the pellets. The lower limit of sensitivity for the rat PRL assay was 5.2 ng/tube, for LH it was 1.3 ng/tube and for FSH it was 30 ng/tube. Intra-assay coefficient of variance for 10 times was as follows: FSH (3.56%), LH (2.64%), and PRL (5.9%). Calculated inter-assay coefficient variances for 10 times were 8.98% for FSH, 7.52% for LH and 5.9%for PRL.

### Radioimmunoassay of serum estradiol and progesterone

Concentrations of serum estradiol were measured using CIS kits (Cisbio Bioassays, France) according to the methods given by the manufacturer. In brief, serum (300 μl) was extracted with 3 ml ethy lether. The ether layer was evaporated under (N2) gas and the extract resuspended in 300 μl of 0.04 M phosphate buffer. After the addition of 100 μl 17/3-estradiol (14000 cpm), each tube was incubated with 100 μl anti-serum raised for 18 hours at room temperature. Goat anti-rabbit r-globulin (1 ml) was subsequently added and the mixture incubated for 15 minutes at room temperature. After centrifugation, the radioactivity in the resulting pellet was counted. The sensitivity was 1.8pg/tube. In order to evaluate serum level of progesterone we mixed serum (0.1 ml), ethylether (1 ml) and propylene glycol (50 μ1). After the ether was evaporated under N2 gas, 0.5 ml phosphate buffer and 0.1 ml (20000 cpm) of iodo-progesterone were added to the tube. The mixture was incubated with 0.1 ml anti-serum raised in rabbits for 18 hours at room temperature. Then, 0.1 ml bovine serum gamma globulin and polyethylene glycol were added to the mixture followed by centrifugation for 10 minutes at 2000 g. We measured radioactivitylevels in the resulting pellet. The lower limit of sensitivity was 0.6 ng/ tube (16). The intra-assay coefficient variances were: 6.12% (for 10 times) for estradiol and 4.8% (for 10 times) for progesterone. Calculated inter-assay coefficients of variance were: estrogen (8.4% for 10 times) and progesterone (9.9% for 10 times).

### Animal mating

In order to evaluate the effect of SPD on natural mating and fertilizing ability in different groups, we randomly chose 5 rats from each group. The chosen rats were adjoined with 1 mature normal male rat for 7 days. The vaginal plugs (white coagulum) were checked every day for 7 days and the vaginal smear process was conducted on cases with vaginal plugs in order to clarify the presence or absence of sperm. The day of sperm detection in smears was considered as day 0 of pregnancy and after 21-23 days (pregnancy period in rats) the numbers of rats that were born were counted.

### Statistical analysis

All results were expressed as mean ± SD. Experimental data were analyzed using analysis of variance and Duncan’s multiple range test (SPSS version 16.00, Chicago, IL, USA). Correlation between the serum levels of PRL with the SPD-administered dosewas analyzed on an Indigo-2 O2 Work Station (Silicon Graphics, Mountain View, CA, USA) using Matlab (MathWorks Inc., Natick, MA, USA). P<0.05 was considered to be statistically significant.

## Results

### Total body weight gain

**Fig 1:**
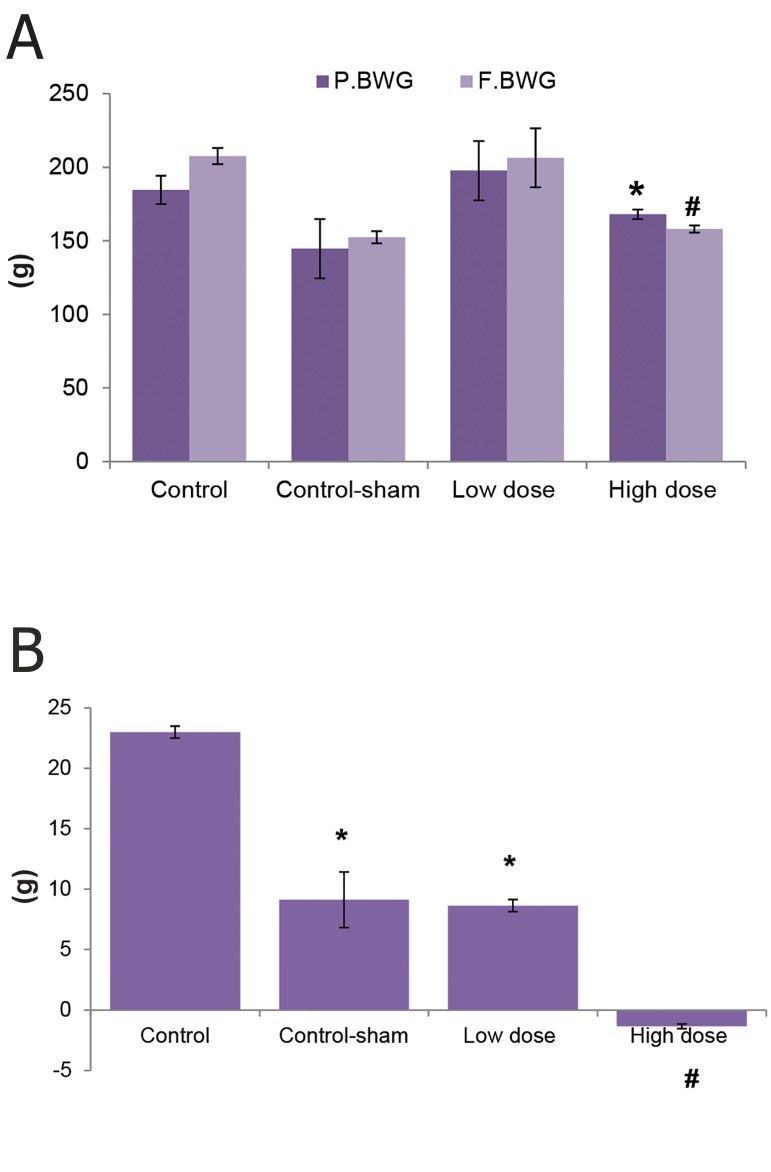
A. Total body weight changes in control, control-sham and test groups. A comparison between primary body weight gain (P.BWG) with final body weight gain (F.BWG) showed that F.BWG remarkably decreased in the high dose sulpiride (SPD) group. B. P.BWG ratio to F.BWG was negative in the high dose SPD-administered group. *;Significant differences (p<0.05) between F.BWG versus P.BWG of the high dose SPD group and #; remarkable differences (p<0.05) between control-sham and low dose SPD groups with high dose SPD group (n=10 for each group). All data are presented as mean ± SD.

We observed no changes in total weight gain of control, control-sham and low dose SPD group sat the end of the treatment period. Animals in high dose SPD group had significantly less weight gain (Fig 1A, B).

### Ovarian follicular growth, atresia and corpus luteum (CL)

**Fig 2:**
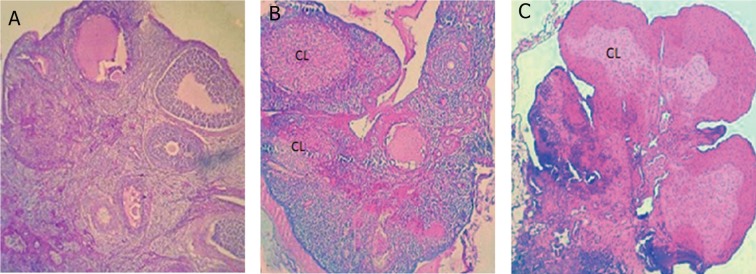
Cross-section from an ovary. A. Control group ovary showed different size follicles and one corpus luteum (CL) that remained from the previous cycle. B. Low dose sulpiride (SPD) group ovary showed sporadic CL and atretic follicles. C. High dose SPD group, note large and active CL without follicular growth. (Hematoxylin-eosin staining, ×400 magnification).

Histological analyses showed that in SPD groups the total number of follicles significantly (p<0.05) decreased in comparison to control and control-sham animals. Both left and right ovaries from the control and control-sham groups contained follicles in various stages of development that included primary, secondary and tertiary follicles of different sizes (<100 ìm to >400 ìm), whereas there was no large antral follicle (>400 ìm) in the high dose SPD group (Fig 2A-C). In SPD-administered animals the cortex of the ovaries (left and right) were covered with small antral atretic follicles (<100-200 ìm). Atresia was mostly present in follicles <100 ìm and additionally observed in 201-300 ìm follicles in high dose SPD rats (Table 1).

**Fig 3:**
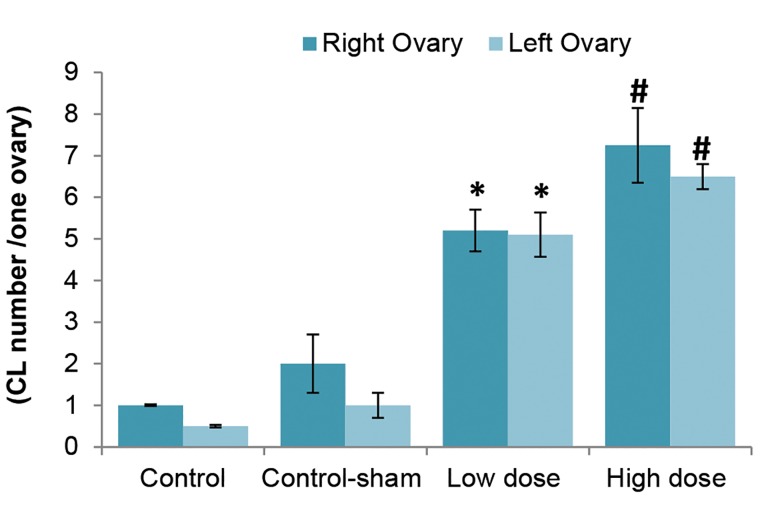
Mean number of corpus luteum (CL) per ovary in different groups. Control and control-sham animals exhibited lower numbers of CLs which remained from previous cycles. Sulpiride (SPD)-dosed animals showed higher numbers of CLs per ovary. *, #; Significant differences (p<0.05) between SPD-administered groups with control and control-sham animals (n=10 for each group). All data are presented as mean ± SD.

Comparing the rate of normal follicles between the control, control-sham and test groups revealed a significant (p<0.05) decrease in the SPD groups. This reduction was dose-dependent; animals that received high dose SPD had the lowest number of normal follicles. The highest rate of normal follicles between the test groups was observed in the low dose SPD group. The data for normal follicles are depicted in table 2. We observed that animals who received SPD exhibited significantly higher numbers of CL compared to the control and control-sham groups (Fig 3).

**Table 1:** Mean numbers of atretic follicles on the right and left ovaries of different study groups. Sulpiride (SPD) administration significantly increased follicular atresia in both the right and left ovaries

	
Atretic follicles of right ovaries					
Groups	<100 μm	101-200 μm	201-300 μm	301-400 μm	>400 μm

**Control**	4.31 ± 1.10	7.57 ± 1.18	12.14 ± 1.80	3.17 ± 0.11	1.43 ± 0.50
**Control-sham**	5.14 ± 1.19	8.14 ± 2.20	13.10 ± 1.10	3.78 ± 3.19	3.30 ± 0.05
**Low dose SPD**	15.75 ± 3.24	10.25 ± 6.60*	3.50 ± 4.43*	1.25 ± 0.95*	1.25 ± 0.89
**High dose SPD**	26.25 ± 2.08*#	23.25 ± 7.41*#	1.50 ± 0.38*#	1.11 ± 0.98*	0.75 ± 0.50*
**Atretic follicles of left ovaries**					
**Control**	3.48 ± 2.80	6.25 ± 1.70	11.19 ± 2.26	2.80 ± 1.00	1.70 ± 0.60
**Control-sham**	6.70 ± 0.05	9.14 ± 1.80	14.16 ± 1.13	14.12 ± 1.10	2.10 ± 0.75
**Low dose SPD**	16.50 ± 2.84*	14.52 ± 4.79	3.75 ± 1.61*	1.75 ± 0.62*	0.25 ± 0.02*
**High dose SPD**	36.33 ± 3.15*#	20.00 ± 6.24*#	1.00 ± 0.70*#	0.00 ± 0.00*#	0.00 ± 0.00*#
	

*; Indicate significant differences (p<0.05) between data of SPD-administered groups with control and control-sham groups and #; Remarkable differences (p<0.05) between low dose SPD group and high dose SPD group. All data are presented as mean ± SD.

**Table 2:** Mean size of normal follicles on right and left ovaries in different groups. Sulpiride (SPD) administration significantly increased normal follicles in both right and left ovaries

	
Normal follicles of right ovaries					
Groups	<100 μm	101-200 μm	201-300 μm	301-400 μm	>400 μm

**Control**	64.12 ± 5.11	21.22 ± 7.32	12.14 ± 11.12	5.11 ± 1.10	4.84 ± 2.20
**Control-sham**	58.34 ± 4.12	18.14 ± 3.16	10.14 ± 3.10	2.48 ± 0.76	3.41 ± 1.80
**Low dose SPD**	36.50 ± 6.60*	7.25 ± 2.34*	4.52 ± 1.69*	0.25 ± 0.48*	0.50 ± 0.24*
**High dose SPD**	18.00 ± 3.46*#	6.26 ± 1.14*	0.50 ± 0.57*	0.20 ± 0.03*	0.5 ± 0.05*
**Normal follicles of left ovaries**					
**Control**	62.43 ± 6.12	22.26 ± 8.14	11.10 ± 2.20	3.40 ± 1.50	3.47 ± 1.57
**Control-sham**	59.12 ± 6.13	17.10 ± 1.00	9.05 ± 1.08	21.11 ± 1.22	3.00 ± 1.01
**Low dose SPD**	29.25 ± 5.91*	8.50 ± 0.72*	2.25 ± 0.23*	0.50 ± 0.05*	0.62 ± 0.47*
**High dose SPD**	20.00 ± 5.57*	9.00 ± 3.00*	1.33 ± 0.30*#	1.30 ± 0.29*#	0.00 ± 0.00*#
	

*; Significant differences (p<0.05) between data of SPD-administered groups with control and control-sham groups and #; Remarkable differences (p<0.05) between low dose SPD group with high dose SPD group. All data are presented as mean ± SD.

### Uterine horn histomorphometry

**Fig 4:**
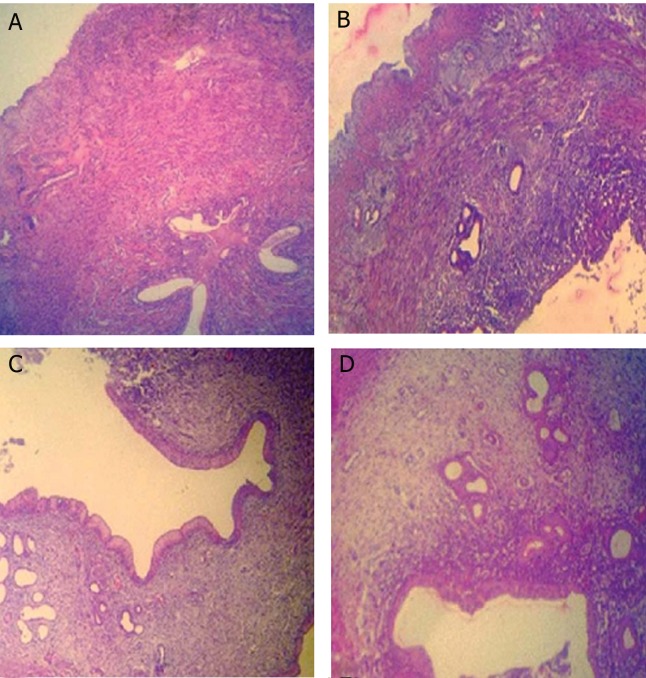
Cross-section from the uterine horn. There are no remarkable histological changes between control. A. And control-sham, B. Animals uterine horns. Normal endometrial thickness and gland distribution is seen in the uterine horns of both groups. C. Low dose sulpiride (SPD) group shows elevated endometrial thickness associated with increased glandular tissue. D. High dose SPD groupshows significantly increased endometrial thickness and gland distribution per mm^2^ of endometrium compared to the other groups. (Hematoxylin-eosin staining, ×600 magnification).

**Fig 5:**
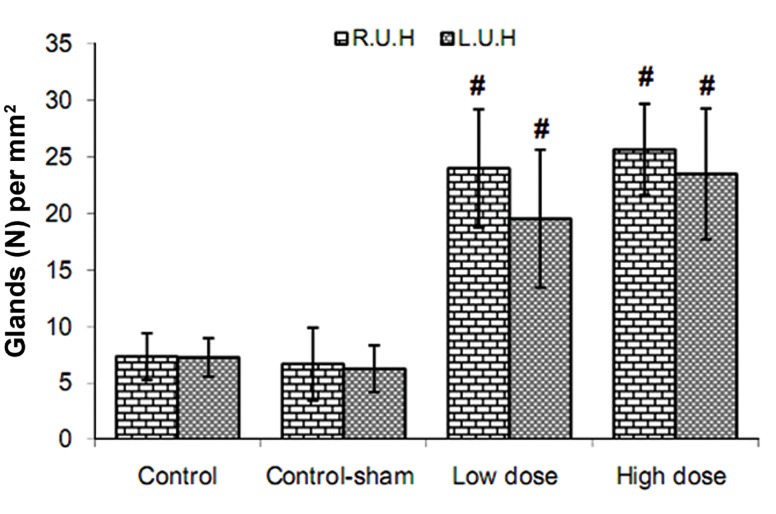
Mean distribution of glands per mm^2^ of uterine endometrium in different groups. Sulpiride (SPD) administration increased gland numbers in the endometrium. #; Significant differences (p<0.05) between SPD-administered groups with control and control-sham animals (n=10 for each group). There are no significant differences (p>0.05) between low and high dose SPD-administered groups. All data are presented as mean ± SD.

Comparing the uterine horn endometrial epithelium height (left and right) between all groups showed that in SPD-administered groups the epithelial height significantly (p<0.05) increased compared to control and control-sham animals. This impairment was SPD dose dependent as the group that received a high dose of SPD showed the highest endometrial epithelium. Simultaneously the endometrial, myometrium and perimetrium thicknesses in both side uterine hornswere remarkably (p<0.05) increased in both the low and high SPD-administered groups (Fig 4A-D). In SPD groups, the numbers of endometrial glands per mm^2^ of the endometrium were considerably (p<0.05) increased. Animals in control and control-sham groups had remarkably lower numbers of endometrial glands per mm^2^ of endometrium (Fig 5). The data for histomorphometric analyses are presented in table 3.

**Table 3:** Histomorphometric data for uterine horns in different groups. Sulpiride (SPD) administration increased uterine horn diameter by elevating endometrial, myometrium and perimetrium thicknesses


Parameters (μm)	Right uterine horns			
	Control	Control-sham	Low dose SPD	High dose SPD

**Uterine horn diameter**	523.62 ± 48.06	529.12 ± 51.29	1245.49 ± 88.29*	1779.93 ± 52.59*#
**Endometrial epithelium height**	10.44 ± 1.72	10.81 ± 1.83	20.12 ± 1.25*	27.44 ± 2.72*#
**Endometrial thickness**	130.22 ± 8.91	123.53 ± 5.01	317.70 ± 10.06*	488.55 ± 9.70*#
**Myometrium thickness**	108.24 ± 4.69	104.04 ± 6.05	231.12 ± 8.59*	291.15 ± 9.21*#
**Perimetrium thickness**	6.13 ± 1.29	5.86 ± 0.96	9.34 ± 2.57*	10.27 ± 2.26*#
**Left uterine horns**				
**Uterine horn diameter**	587.31 ± 31.50	600.10 ± 21.32	1351.64 ± 29.52*	1801.21 ± 23.64*#
**Endometrial epithelium height**	10.55 ± 3.90	12.71 ± 1.39	18.67 ± 3.77*	30.44 ± 5.03*#
**Endometrial thickness**	138.12 ± 9.19	128.70 ± 60.70	438.99 ± 9.65*	528.96 ± 13.10*#
**Myometrium thickness**	110.47 ± 3.61	101.50 ± 6.13	193.28 ± 13.94*	244.75 ± 16.74*#
**Perimetrium thickness**	7.00 ± 1.05	6.18 ± 2.00	10.91 ± 1.18*	17.49 ± 7.14*#


*; Significant differences (p<0.05) between data of SPD-administered groups with control and control-sham groups and #; Remarkable differences (p<0.05) between low dose SPD group with high dose SPD group. All data are presented as mean ± SD.

### Mammary gland morphometry

**Fig 6:**
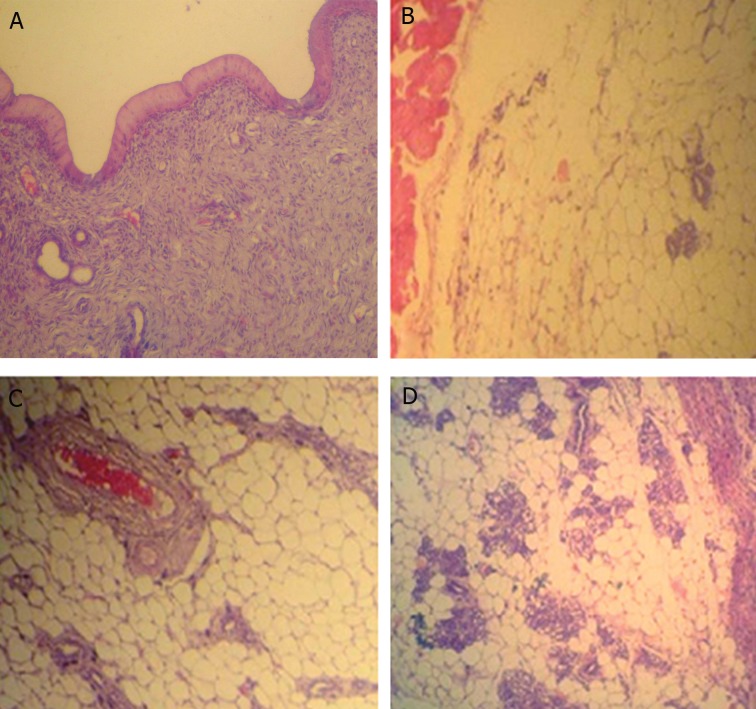
Cross-section from mammary gland. Note inactive mammary glands in control. A. And control-sham, B. groups. Developed secretory alveoli and ducts are presented in low dose, C. and high dose, D. Sulpiride (SPD)-administered groups. The lactating alveolus diameter in the glands and the distribution of lactiferous ducts considerably increased in SPD-administered animals. (Hematoxylin-eosin staining, ×400 magnification).

Light microscopic analyses showed that the lactating alveolus diameter in glands and the distribution of lactiferous ducts remarkably (p<0.05) increased in SPD-administered groups. The alveolar epithelium height significantly increased in animals treated with SPD, which was dose dependently increased in SPD-treated animals. Histological analyses showed that simultaneous with remarkable secretory alveoli and ductal development,there were fatty globules observed in the secretory and intra-lobular ducts of the SPD-administered rats’ mammary glands (Fig 6A-D). The glands of the control and control-sham animals had inactive lobules and secretory ducts. The data for histomorphometric analyses are shown in table 4.

### Serum levels of PRL, LH, FSH, estradiol and progesterone

**Fig 7:**
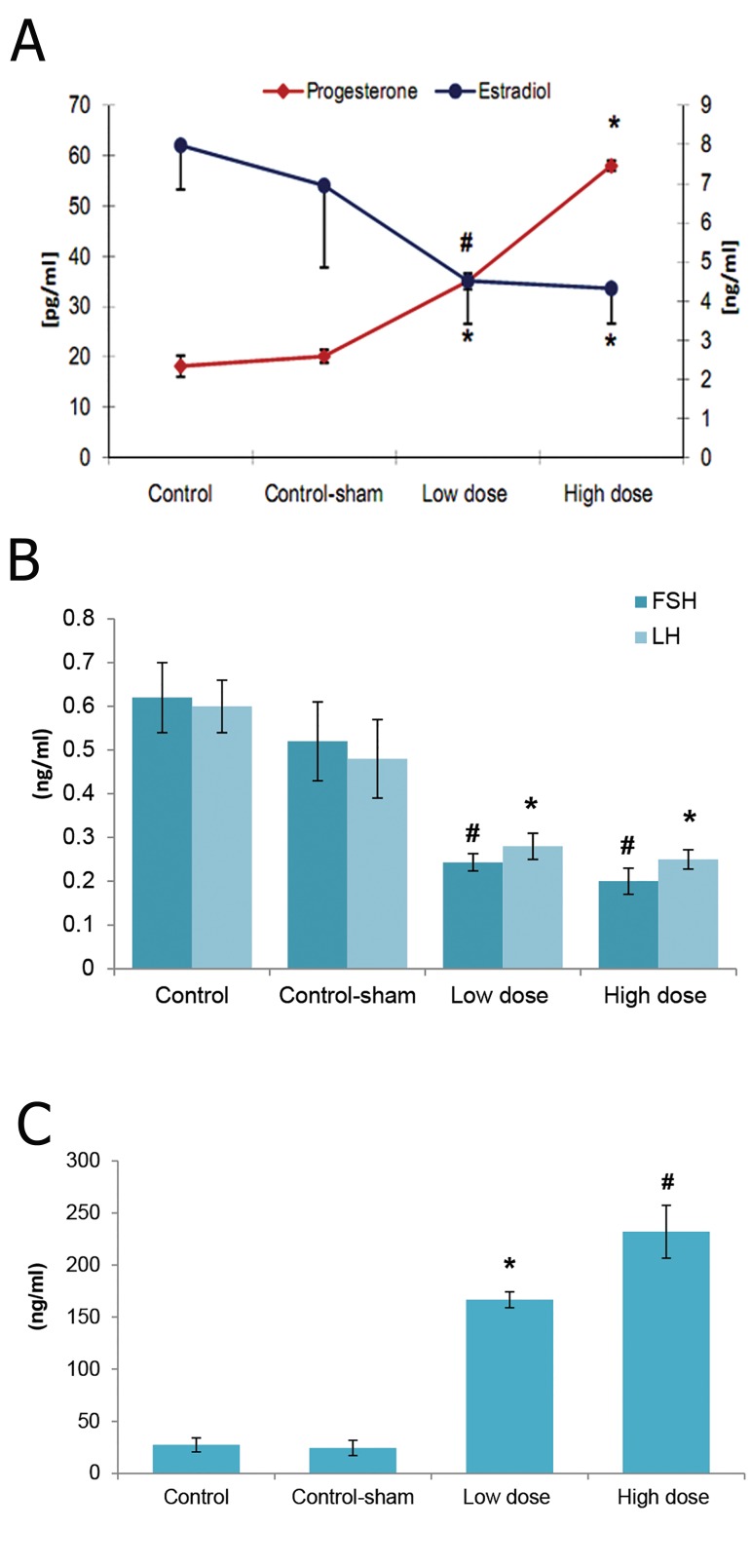
Mean serum levels of estradioland progesterone A. luteinizing hormone (LH) andfollicle stimulating hormone (FSH) B. and prolactin (PRL) C. in different groups. Marked data indicate significant differences (p<0.05) between low and high dose sulpiride (SPD) groups (n=10 for each group) with control and control-sham groups (n=10 for each group). There are remarkable differences (p<0.05) between serum levels of progesterone and PRL of low and high dose SPD groups. All data are presented as mean ± SD. *, #; Significant (p<0.05) difference between marked groups with each other and with control and control-sham groups.

**Fig 8:**
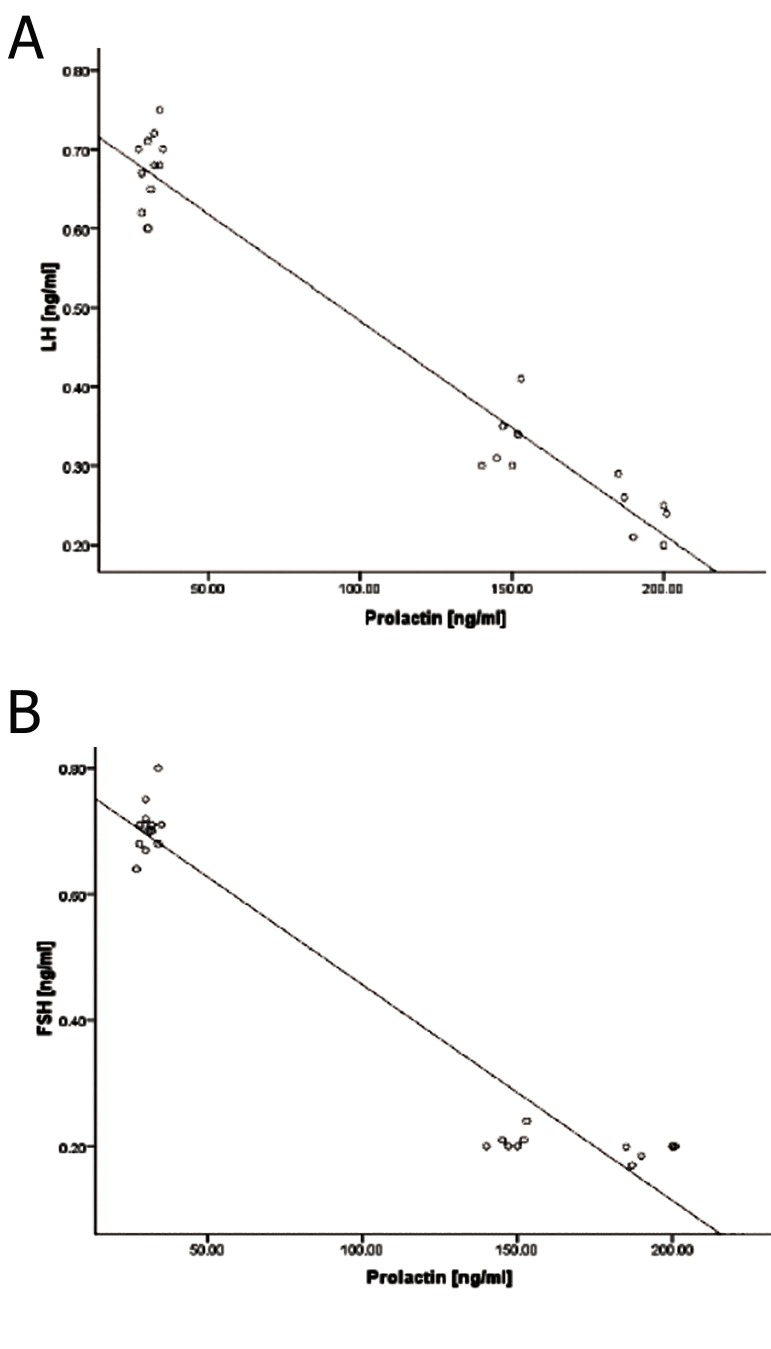
The correlation between prolactin (PRL) levels with luteinizing hormone (LH). A. and between PRL and follicular stimulating hormone (FSH). B. in different groups (n=10 for each group). There are negative correlations between the serum levels of PRL with LH (r2=0.974) and FSH (0.969).

Biochemical analyses showed that the serum levels of PRL significantly (p<0.05) increased in animals that received low and high doses of SPD. In contrast, control and control-sham animals showed constant PRL levels. A comparison of LH and FSH serum levels between SPD and control groups showed that serum LH and FSH levels remarkably (p<0.05) decreased in animals that received either low or high doses of SPD. Although reduction in LH and FSH levels progresse daccording to SPD administration doses, we observed no statistically significant (p>0.05) difference between low and high dose SPD-administered animals. The serum levels of estradiol significantly decreased, while the progesterone level remarkably increased in animals treated with SPD. The high dose SPD-administered group showed the lowest serum level of estradiol and the highest level of progesterone compared to the low dose SPD, control and control-sham groups. The data for hormonal analyses are presented in Figures 7A-C. The data for correlation between PRL, LH and FSH are presented in Figures 8-A and 8-B.

**Table 4:** Histomorphometric data for mammary gland lobules in different groups. Sulpiride (SPD) administration increased the lobular diameter, which was dose dependent


Mammary glands			
Parameters (μm)	Control Control-sham	Low dose SPD	High dose SPD

Lobular diameter	47.85 ± 3.13	48.42 ± 4.22	135.88±16.39*	251.35±29.28*#


*; Significant differences (p<0.05) between data of SPD-administered groups with control and control-sham groups and #; Remarkable differences (p<0.05) between low dose SPD group with high dose SPD group. All data are presented as mean ± SD.

### Fertilizing indexes and neonates

Analysis of the fertilizing index in the control, control-sham and SPD test groups showed that the SPD-administered groups had a negative fertilizing index with no neonates. In contrast the control and control-sham animals showed positive fertilizing indexes with 31 and 19 neonates, respectively (Table 5).

**Table 5:** Fertilizing index (pregnant rats) and number of neonates after animals were adjoined. Sulpiride (SPD) administration lowered the fertilizing index to zero. There were no neonates observed in the SPD groups


Parameters (μm)	Control	Control-sham	Low dose SPD	High dose SPD

**Rats (n)**	4	4	4	4
**Fertilizing index**	100	75	0	0
**Neonates (n)**	31	19	0	0


*; Significant differences (p<0.05) between data of SPD-administered groups with control and control-sham groups and #; Remarkable differences (p<0.05) between low dose SPD group with high dose SPD group. All data are presented as mean ± SD.

## Discussion

In the present study we used SPD (antipsychotic drug) to analyze the SPD-induced hyperprolactinemia effect on the development and consecutive atresia of different sized follicles and investigated the histological alterations in uterine horns and mammary glands. The obtained results revealed that SPD elevated serum PRL concentration. The increased PRL level correlated with alterations in serum LH and FSH levels. The plasma levels of estradiol and progesterone significantly changed. SPD-induced ovaries exhibited sporadic follicular atresia in different sizes. The uterine horns had significantly increased wall thicknesses and the mammary glands were manifested with galactorrhea features.

It is well established that PRL secretion is regulated via secretion of dopamine in the tubero- infundibular tract (4). Dopamine is known as a PRL-inhibiting factor on D2 receptors (3, 17) which are located on the surface of the pituitary lactotroph cells. Conventional antipsychotic drugs block the D2 receptors and in turn induced impairment results in the loss of inhibitory effects of dopamine (17). The results from our biochemical analyses support the mentioned hypothesis; accordingly, serum PRL levels have significantly (p<0.05) increased in SPD-administered groups which were SPD dose-depended. Therefore, we have concluded that SPD not only could exert hyperprolactinemia but also induced hyperprolactinemia is enhanced according to the dose administered.

It is well known that PL inhibits follicular estradiol production (9, 13, 18). In rodents, estrogen is essential for follicular growth, differentiation and for preventing preantral and early antral follicle apoptosis in rats (19). Histological observations have demonstrated that SPD-administered animals exhibit remarkably increased atresia (different sizes); these ovaries had higher numbers of CL. On the other hand, according to the dose, the serum level of estrogen decreased with simultaneous increase in progesterone concentration in SPD-administered groups. Thus, increased PRL levels with a synergistic effect of progesterone resulted in remarkable follicular atresia. Of note, smaller size follicles at the high dose and larger follicles at the low dose in SPD-administered animals were remarkable for atresia which suggested that the adverse effect (atresia) exerted by SPD was dependent on the administered dose. These impairments might not only attributeto a higher level of PRL butthey might attribute to CL resistance from previous cycles that in turn lead to severe follicular atresia, which did not allow estradiol secretion to restart. The reduced serum level of estradiol in SPD-administered animals proved the above theory.

According to earlier findings and as previously indicated, the increased level of PRL can largely affect gonadotropins. Our analyses have shown that serum levels of LH and FSH significantly decreased in both low and high dose SPD-administered groups. In patients who have been treated with antipsychotic-drugs, reduced synthesis and secretion of the GnRH in the hypothalamus is able to decrease enough stimulation for LH and FSH secretion in the pituitary gland (20). Therefore, we can conclude that, primarily, SPD blocked the hypothalamus-pituitary axis which in turn inhibited gonadotropin secretion. Secondly, positive feedback of estradiol through the pituitary gland for LH hormone secretion was eliminated. Thus the serum levels of LH and FSH decreased significantly in SPD-administered animals (Fig 8). Additionally, the decreased estradiol level associated with reduced gonadotropins resulted in CL resistance which was delivered from the previous cycle and ultimately enhanced atresia in SPD-administered animals. Inhibited follicular growth (marked with reduced normal follicles) in the SPD-induced groups proved this theory. In order to evaluate the biological activity of CLs, we investigated the serum levels of progesterone. Observations demonstrated that the serum level of progesterone remarkably increased in SPD-administered animals. This finding showed that the observed CLs were considerably active. Additionally, because of increased levels of progesterone and absence of an appropriate feedback for androgens and estradiol secretion, in order to start a new cycle (21, 22), follicular growth depression occurred in the ovaries of SPD-administered animals.

Light microscopic observations demonstrated significant increases in endometrial thickness and glandular structure of the endometrium in both low and high dose SPD-administered groups. By considering the luteotrophic effect of PRL and the simultaneous increased progesterone level, we hypothesized that following an increased level of PRL and accomplishing this impairment with higher concentration of progesterone, there was an increase in endometrial thickness and gland distribution in SPD-administered animals. Accordingly the animals that received high dose SPD showed remarkably higher gland numbers per mm^2^ of endometrium.

**Fig 9:**
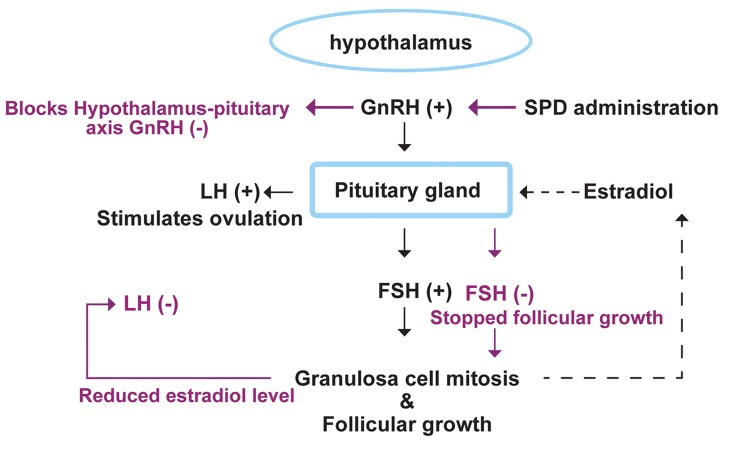
Regulatory mechanism involved in follicle stimulating hormone (FSH) and luteinizing hormone (LH) secretion and sulpiride (SPD) effect. SPD blocks GnRH secretion which in turn leads to severe reductions in serum levels of FSH and LH.

Mammary gland alveolar development, alveolar epithelium proliferation and/or differentiation largely depend on PRL hormone stimulation (20, 23). Histological analyses showed increased fatty globules in mammary glands and intra-lobular ducts of the high dose SPD-administered rats. These features have shown that the intensity of galactorrhea majorly depends on the PRL level which remarkably increased in the high dose SPD-administered group. From the above mentioned findings, we have concluded that by reduced follicular growth the ovulation ratio will decrease severely which in turn leads to a negative fertilizing index in SPD-administered animals which was proven by our results of the fertilizing indexes in the different groups. Accordingly animals in the SPD-induced groups had with negative fertilizing indexes and there were no neonates following adjoining the animals with normal male rats (Fig 9).

## Conclusion

Our results have shown that although SPD is widely used as an antipsychotic drug, the rats treated with low and high doses of SPD had mean serum PRL levels that might be several-fold greater than the upper limit of normal. Administrated dose of SPD the prevalence of hyperprolactinemia. Additionally the SPD-induced hyperprolactinemia was associated with a disturbance in the levels of essential reproductive hormones, estradiol and progesterone. The PRL-associated disturbances in gonadotropins and reproductive hormones exerted significant adverse effects on follicular growth and resulted in galactorrhea features in SPD-administered rats. Ultimately SPD, as a PRL-elevating antipsychotic drug, decreased the fertilizing index especially at the higher dose. All the above mentioned results were remarkably SPD dose-dependent.
